# Collagen recognition and transmembrane signalling by discoidin domain receptors^☆^

**DOI:** 10.1016/j.bbapap.2012.10.014

**Published:** 2013-10

**Authors:** Federico Carafoli, Erhard Hohenester

**Affiliations:** Department of Life Sciences, Imperial College London, London SW7 2AZ, UK

**Keywords:** DDR, discoidin domain receptor, DS, discoidin, ECM, extracellular matrix, JM, juxtamembrane, mAb, monoclonal antibody, RTK, receptor tyrosine kinase, TM, transmembrane, Receptor tyrosine kinase, Collagen, X-ray crystallography

## Abstract

The discoidin domain receptors, DDR1 and DDR2, are two closely related receptor tyrosine kinases that are activated by triple-helical collagen in a slow and sustained manner. The DDRs have important roles in embryo development and their dysregulation is associated with human diseases, such as fibrosis, arthritis and cancer. The extracellular region of DDRs consists of a collagen-binding discoidin (DS) domain and a DS-like domain. The transmembrane region mediates the ligand-independent dimerisation of DDRs and is connected to the tyrosine kinase domain by an unusually long juxtamembrane domain. The major DDR binding site in fibrillar collagens is a GVMGFO motif (O is hydroxyproline), which is recognised by an amphiphilic trench at the top of the DS domain. How collagen binding leads to DDR activation is not understood. GVMGFO-containing triple-helical peptides activate DDRs with the characteristic slow kinetics, suggesting that the supramolecular structure of collagen is not required. Activation can be blocked allosterically by monoclonal antibodies that bind to the DS-like domain. Thus, collagen most likely causes a conformational change within the DDR dimer, which may lead to the formation of larger DDR clusters. This article is part of a Special Issue entitled: Emerging recognition and activation mechanisms of receptor tyrosine kinases.

## Highlights

► DDR1 and DDR2 are receptor tyrosine kinases that are activated by collagen. ► DDR activation by collagen is slow and sustained. ► The principles of collagen recognition are understood from structural studies. ► The mechanism of transmembrane signalling is currently not understood. ► Possible activation mechanisms are discussed.

## 1 Introduction

Receptor tyrosine kinases (RTKs) are a large family of transmembrane proteins that consist of a variable ligand-binding extracellular region (ectodomain), a single membrane-spanning region, and a conserved cytosolic tyrosine kinase domain. Signalling by RTKs critically controls many fundamental cellular processes, such as proliferation, differentiation, survival and migration [1,2]. The human genome encodes 58 RTKs, which are divided into 20 subfamilies according to the modular architecture of their ectodomains [1]. The subject of this review is the discoidin domain receptor (DDR) subfamily, which in vertebrates has two members, DDR1 and DDR2. In particular, we focus on the structural basis of ligand recognition by DDRs and how they might become activated following ligand binding. The downstream signalling pathways and the roles of DDRs in human disease are discussed in several recent reviews [3–5].

The DDRs were discovered by homology cloning in searches for novel RTKs [6–12]. The defining feature of the DDRs is the presence of a discoidin (DS) domain in their extracellular region. The DS domain was originally described in the discoidin I protein of the slime mold *Dictyostelium discoideum* [13]; an alternative name for the DS domain is factor 5/8 type C domain due to the presence of homologous domains in the blood coagulation factors V and VIII [14,15]. Another feature of the DDRs that was noticed immediately is that they contain an unusually long cytosolic juxtamembrane (JM) region linking the transmembrane (TM) region to the tyrosine kinase domain. The most atypical property of the DDRs, however, turned out to be the nature of their activating ligand (which was unknown when the receptors were cloned). The prototypical RTKs, exemplified by the epidermal growth factor receptor, are activated by small soluble proteins and a similar situation might have been expected for the DDRs. The search for the DDR ligand was carried out independently by two laboratories and yielded the surprising result that the DDRs are activated by collagen [16,17], a major component of the vertebrate extracellular matrix (ECM) [18]. Vogel et al. found that cells expressing DDR1 showed robust receptor phosphorylation when Matrigel, a mixture of ECM proteins secreted by Engelbreth–Holm–Swarm mouse sarcoma cells, was added to the culture medium [17]. Shrivastava et al. used DDR-Fc fusion proteins to isolate from the surface of cancer cells a large DDR-binding protein that was characterised by a high content of glycine, proline and hydroxyproline [16]. From these initial observations, both teams went on to identify collagen as the DDR ligand, thus attributing for the first time a direct role for the ECM in RTK signalling [16,17]. Until then, the ECM had been believed to contribute to RTK signalling only indirectly by capturing and storing growth factors [19], and a signalling role of collagen had only been described for integrins and the platelet receptor, glycoprotein VI [4,20,21]. Importantly, DDR activation by collagen was shown to be independent of β1 integrins [22]. Both DDRs are activated by fibrillar collagens (mainly types I, II and III), whilst their preferences for non-fibrillar collagens are distinct, with DDR1 and DDR2 being specific for type IV and type X collagen, respectively [16,17,23]. DDR activation strictly requires collagen to be in its native, triple-helical, conformation; heat-denatured collagen (gelatin) is not recognised by the DDRs [16,17].

Ligand binding to prototypical RTKs results in the autophosphorylation of specific cytosolic tyrosine residues within seconds to minutes of stimulation, followed by the rapid down-regulation of the signal [1,2]. In contrast, treatment of DDR-expressing cells with collagen results in slow and sustained DDR phosphorylation that lasts up to several hours [16,17]. The mechanism underlying this slow activation kinetics is unknown and should be studied with urgency.

The biological outcomes of DDR activation and the role of DDRs in human diseases are only partly understood. The DDRs are widely expressed in mammalian tissues and regulate cell adhesion, migration, proliferation and differentiation [3,4], as well as the remodelling of the ECM by matrix metalloproteinases [24,25]. Mice lacking DDR1 or DDR2 are viable, but display impaired growth and a variety of other defects. The impaired skeletal growth in *Ddr2*−/− mice is due to reduction in chondrocyte proliferation [26] and mirrored by a rare form of dwarfism in humans resulting from mutations in the *DDR2* gene, termed spondylo-meta-epiphyseal dysplasia short limb-hand type [27,28]. The phenotype of the *Ddr1*−/− mice includes female infertility, as well as defects in the mammary gland [29], kidney [30] and inner ear [31]. Mice with a spontaneous deletion that removes most of the *Ddr2* gene (*slie* mice) are infertile due to a gonadal dysregulation [32]. Aberrant DDR function in humans is implicated in fibrotic disorders of several organs, atherosclerosis, arthritis, and various cancers [3–5]. Similarly to other RTKs, the DDRs are recognised as potentially valuable therapeutic targets [5].

## 2 Primary sequence and domain organisation of DDRs

**Fig. 1:**
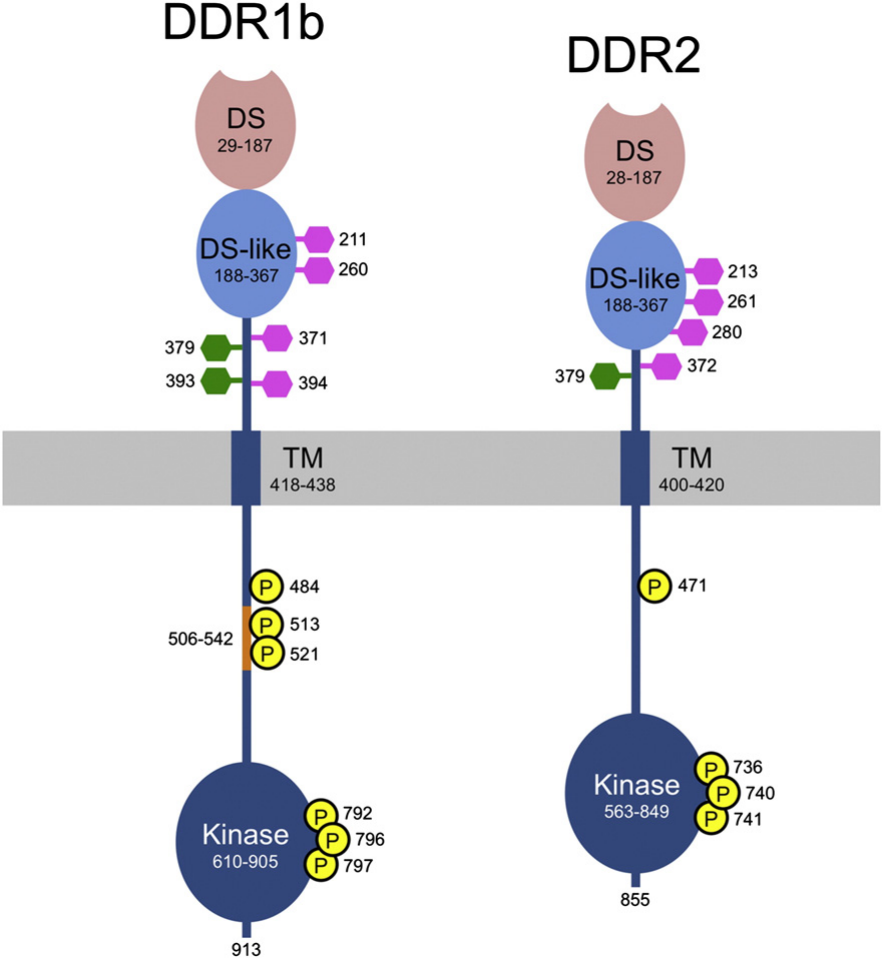
Schematic representations of human DDR1b and DDR2. The domain boundaries are indicated for the discoidin (DS) domain, the DS-like domain, the TM region, and the tyrosine kinase domain. The cell membrane is represented by a grey bar. The DDR1a splice variant lacks residues 506–542 (shown in orange). Predicted *N*- and *O*-linked glycosylation sites are indicated by magenta and green hexagons, respectively. Biochemically verified phosphorylation sites are shown in the cytoplasmic juxtamembrane regions and the activation loop of the tyrosine kinase domains. The overall sequence identity between human DDR1b and DDR2 is 52.8% (58.9% in the DS domain, 50.8% in the DS-like domain, 68.2% in the kinase domain).

Invertebrates have DDR genes, but whether the encoded proteins function as collagen receptors is not known [4]. All vertebrates have two DDRs, with a typical sequence identity of ~ 50% between DDR1 and DDR2. The human DDR1 gene has 17 exons that are alternatively spliced to generate five DDR1 isoforms (a–e) that differ in the cytoplasmic region [10,33]. DDR1a and DDR1b are the most widely distributed isoforms [3]. DDR1a lacks a 37-residue segment in the cytosolic JM region of DDR1b that contains important phosphorylation sites. The d and e isoforms lack all or a part of the tyrosine kinase domain and are predicted to be inactive. The human DDR2 gene has 19 exons and encodes a single isoform. The extracellular regions of DDRs consist of an N-terminal DS domain that contains the collagen-binding site (~ 160 residues) [34,35], followed by a recently described DS-like domain (~ 180 residues) [36], and an extracellular JM region that is poorly conserved between DDR1 and DDR2 (50 residues in human DDR1, 32 residues in human DDR2) (Fig. 1). *N*-linked glycosylation sites are present in the DS-like domain and the extracellular JM region, which additionally contains several predicted *O*-linked glycosylation sites. Following the TM region, there is a large cytosolic JM region (170 residues in DDR1b and DDR1c, 143 residues in DDR1a, 142 residues in DDR2) and, finally, the tyrosine kinase domain. The cytoplasmic tails of DDRs are unusually short and devoid of tyrosine residues.

Crystal structures have been determined of the DS/DS-like tandem of DDR1 in complex with an antibody Fab fragment [36] and of the DS domain of DDR2 bound to a collagen-like peptide [37]. There also exists a solution structure of the free DDR2 DS domain [38]. These structures are described in the following paragraphs. No experimental structures are currently available of the tyrosine kinase domains of DDR1 or DDR2, but their homology to tyrosine kinase domains of known structure allows them to be modelled. Homology models based on the tyrosine kinase domains of c-Abl, insulin receptor and MuSK have been used to explain the inhibition of DDRs by imatinib, nilotinib and dasatinib [39]. To what extent the unusually long JM regions of DDRs are structured is unknown and an important question for further study.

## 3 The DS domain: structural basis of collagen binding

**Fig. 2:**
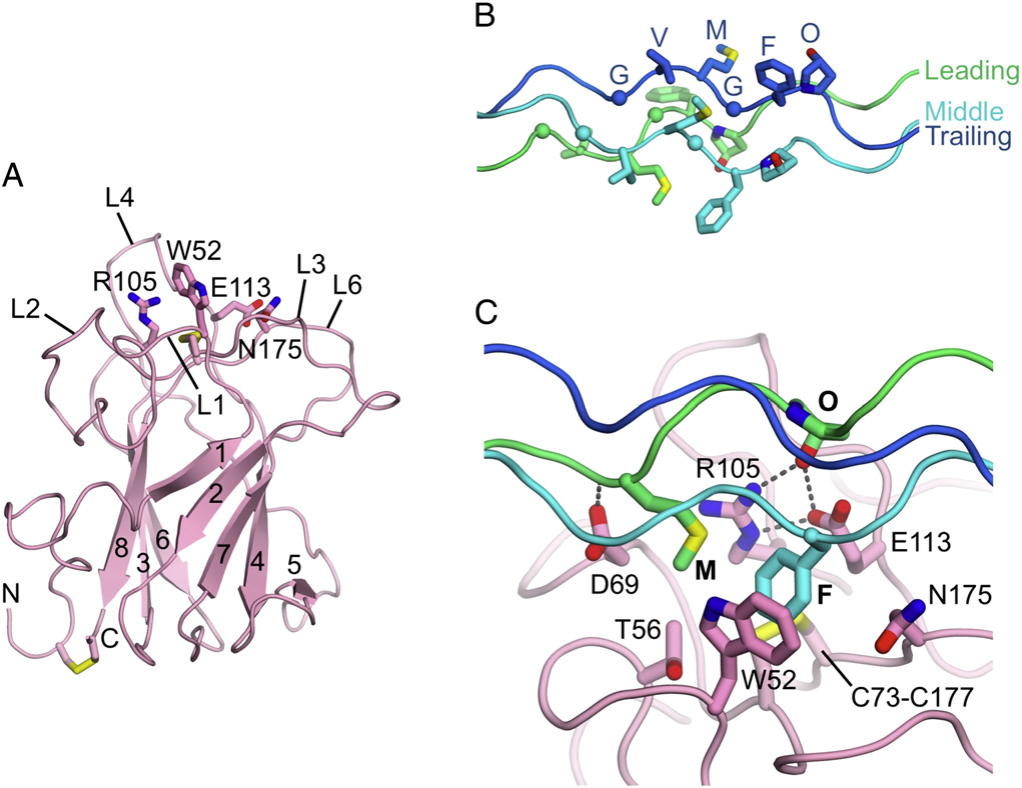
Structures of the DDR2 DS domain and its collagen complex. (A) Solution structure of the DS domain of human DDR2 (PDB code 2Z4F) [38]. The eight β-strands are labelled 1-8 and the N- and C-termini are indicated. The loops at the top of the DS domain are labelled L1–L4 and L6. Disulphide bridges are shown as sticks with yellow sulphur atoms. Selected residues involved in collagen binding (Trp52, Arg105, Glu113, and Asn175) are shown as sticks and are labelled. (B) Structure of the GVMGFO motif (O is hydroxyproline) in the homotrimeric collagen peptide used for co-crystallisation (PDB code 2WUH) [37]. Note the one-residue stagger between chains, which are referred to as the leading, middle and trailing chains [42]. The amino acids of the GVMGFO motif are labelled in the trailing chain. (**C**) Close-up view of the collagen-binding site in the crystal structure of the DS domain of human DDR2 bound to the GVMGFO-containing collagen peptide (PDB code 2WUH) [37]. The methionine of the collagen leading chain (M) and phenylalanine of the middle chain (F) are accommodated in a trench at the top of the DS domain, between Trp52 and the Arg105-Glu113 salt bridge. The hydroxyproline of the collagen leading chain (O) forms hydrogen bonds with the Arg105-Glu113 salt bridge (shown as dashed lines).

Mutagenesis experiments based on homology models of the DDR DS domain established that several predicted loop regions are essential for collagen binding and receptor activation [35,40]. The solution structure of the DDR2 DS domain [38] confirmed the predicted factor 5/8 type C fold, which consists of an eight-stranded β-barrel arranged in two antiparallel β-sheets (β1–β2–β7–β4–β5 sheet and β6–β3–β8 sheet) (Fig. 2A). At the bottom of the β-barrel, the β2–β3 and β6–β7 connections form a flat surface, and the N- and C-termini of the DS domain are held together by a strictly conserved disulphide bridge [14,15]. The top of the barrel is characterised by five protruding loops, a common feature of DS domains, with loops L1–L3 connecting strands β1 and β2, L4 connecting β3 and β4, and L6 connecting β7 and β8. According to the earlier mutagenesis studies, loops L1, L2 and L4 are involved in collagen binding to DDR2 [35]. The footprint of collagen II on DDR2 was mapped by transferred cross-saturation experiments [38] and identified the following residues as important for collagen binding: Trp52 (L1); Arg105, Ile112 and Glu113 (L4); Met174 and Asn175 (L6); and the Cys73-Cys177 disulphide bridge at the bottom of the trench formed by loops L1–3, L4 and L6 (Fig. 2A). A generic collagen triple helix was docked computationally to this footprint, but this model did not reveal the specific roles of the identified DDR2 residues in collagen recognition [38].

Mapping the DDR-binding sites on collagen proved challenging due to the large size and insoluble nature of this structural ECM protein. The fibrillar collagen types I–III consist of three polypeptide chains of ~ 1000 residues, termed α chains, that form a right-handed triple helix with a one-residue stagger between chains. The α chains have a characteristic sequence of repeating glycine–X–Y residues, with positions X and Y often occupied by proline and 4-hydroxyproline, respectively [18,41]. Type I collagen is a heterotrimer consisting of two types of α chains, whereas collagen types II and III are homotrimers. Because of the one-residue stagger between chains, equivalent residues in different chains of a collagen homotrimer have non-equivalent chemical environments. The three chains are commonly referred to as the leading, middle and trailing chains [42].

The first attempt to map DDR-binding sites in collagen was made using atomic force and transmission electron microscopy [43]. These experiments visualised DDR2 bound to the triple helix of collagen type I, but did not identify specific binding sites. A specific DDR2-binding site within the D2 period of type II collagen (residues 235–468 of the triple-helix) was identified using recombinant collagen II variants, but a more detailed characterisation of the binding site was not possible using these reagents [44]. A systematic analysis of DDR-collagen interactions was made possible by the development of the Collagen Toolkits, which are libraries of overlapping triple-helical peptides that collectively represent the entire triple helix of homotrimeric collagens [45]. Experiments with the collagen II Toolkit revealed three major binding sites for DDR2 in type II collagen: triple helix residues 217–243, 397–405 and 775–801 [46]. The central site, which is located within the D2 period previously identified to be important [44] and conserved in type III collagen, was investigated in detail. The use of truncated and alanine-substituted peptides established that the minimal DDR2 binding sequence in the central site is GVMGFO (O is hydroxyproline; Fig. 2B) [46]. Remarkably, this motif overlaps with the binding sites for two other, structurally unrelated, proteins: von Willebrand factor, a plasma protein whose interaction with collagen contributes to haemostasis [47,48]; and SPARC, a small glycoprotein that modulates collagen assembly [49,50]. The functional relevance (if any) of DDRs, von Willebrand factor and SPARC sharing a binding site in collagen is unclear.

The identification of a minimal DDR2-binding motif allowed the design of a triple-helical collagen-like peptide for co-crystallisation with the DS domain. The resulting crystal structure [37] revealed that the DDR2 DS domain interacts with two of the three GVMGFO motifs in the collagen-like peptide. In accordance with the collagen footprint determined previously [38], the triple helix is bound in a trench that is created by the protruding L1, L2, L4 and L6 loops at the top of the DS domain (Fig. 2C). One wall and the floor of the trench are delineated by apolar residues (Trp52, Thr56, Asn175, Cys73–Cys177), whilst the other wall is made up of a salt bridge between Arg105 and Glu113, and the side chain of Asp69. The amphiphilic trench binds the methionine of the leading chain and the phenylalanine of the middle chain, and the Arg105-Glu113 salt bridge forms two hydrogen bonds with the hydroxyl group of the hydroxyproline of the leading chain. These interactions are critical, as shown by the lack of DDR2 binding to peptides in which either M, F or O of the GVMGFO motif is replaced by alanine [46]. Additional interactions, some of them mediated by water molecules, are made with main chain atoms of the collagen-like peptide [37]. By revealing that the major binding determinants are contributed by two chains of the triple helix, the structure explains why denatured collagen is not recognised by DDRs [16,17].

In contrast to DDR2, which binds to several Toolkit peptides derived from type II and type III collagens, DDR1 binding is restricted to the GVMGFO motif [51]. The residues forming the collagen-binding trench are strictly conserved in DDR1 and DDR2, indicating a shared recognition mode of the GVMGFO motif [37]. GVMGFO-containing peptides induce DDR1 and DDR2 phosphorylation (further discussed below) [46]. In contrast, none of the other binding sites observed for DDR2 activate the receptor [51], and GVMGFO thus may be the only functionally relevant DDR-binding motif in fibrillar collagens. Residues at the periphery of the collagen-binding trench are less well conserved between DDR1 and DDR2, and these differences plausibly account for the different specificities of DDRs towards non-fibrillar collagens. Indeed, the substitution of five peripheral amino acids in DDR2 with their DDR1 counterparts converts DDR2 into a receptor for type IV collagen [51]. The DDR1 binding site(s) in type IV collagen remain to be identified.

The affinity of a single DS domain for collagen is modest. Ichikawa et al. used surface plasmon resonance to determine a dissociation constant of 25 μM for the binding of the DDR2 DS domain to immobilised type II collagen [38]. In solid-phase assays with immobilised collagen, monomeric DDR constructs showed no detectable binding, whereas dimeric DDR constructs bound with apparent dissociation constants in the low nanomolar range [35]. Avidity effects are well known to amplify the binding strength in solid-phase assays, but it is also possible that the two DS domains in a DDR dimer co-operate in collagen binding (see below).

The structures of the free DS domain of DDR1 (as seen in the context of the ectodomain structure [36]) and the collagen-bound DS domain of DDR2 [37] are very similar, even in the collagen-binding loops (r.m.s. deviation of 0.57 Å for 145 aligned Cα atoms). This situation contrasts with collagen binding to integrins, which is accompanied by large-scale conformational changes in the receptor that are linked to the process of transmembrane signalling [42,52].

## 4 The DS-like domain

**Fig. 3:**
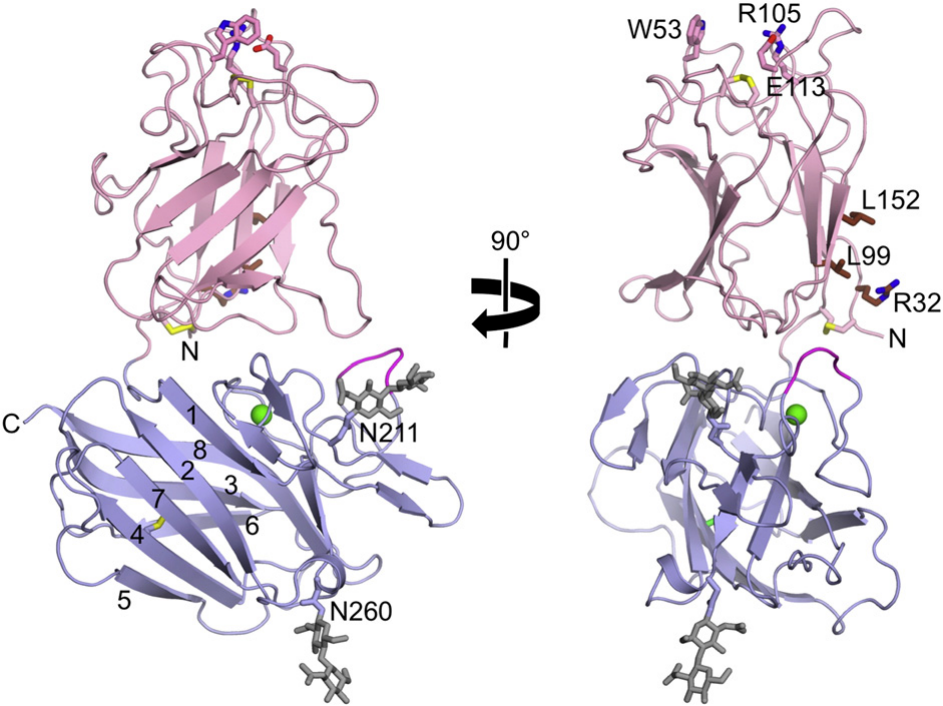
Structure of the ectodomain of DDR1. Shown are two orthogonal views of the human DDR1 ectodomain lacking the juxtamembrane region, as seen in a co-crystal structure with a monoclonal antibody Fab fragment (PDB code 4AG4) [36]. The DS domain is in light pink and the DS-like domain in light blue. The eight core β-strands of the DS-like domain are labelled 1–8 and the N- and C-termini are indicated. The Fab fragment, which is not shown for clarity, is bound near the C-terminus to the β3 strand and the β6-β7 loop of the DS-like domain. Disulphide bridges are shown as sticks with yellow sulphur atoms. A calcium ion is shown as a green sphere. Two *N*-linked glycans are shown in grey and the modified asparagines are labelled. Residues involved in collagen binding (Trp52, Arg105, and Glu113) or required for DDR signalling (conserved patch: Arg32, Leu99, and Leu152) are shown as sticks and are labelled. A protruding loop in the DS-like domain (see text) is highlighted in magenta.

The recent structure determination of the near-complete DDR1 ectodomain bound to the Fab fragment of an inhibitory monoclonal antibody (mAb) revealed that the domain following the DS domain adopts a similar eight-stranded β-barrel fold (r.m.s. deviation of 3.1 Å for 121 aligned Cα atoms), despite a sequence identity of only 9% [36]. To reflect this distant relationship, the second DDR domain was termed the DS-like domain. The β-barrel of the DS-like domain is elaborated by a long insertion between strands β1 and β2, containing five additional strands, two *N*-linked glycosylation sites and a calcium-binding site (Fig. 3). The DS-like domain lacks the disulphide bond that links the N- and C-termini of DS domains, but contains three conserved cysteines in other locations. Two cysteines (Cys303 and Cys348 in DDR1) form an internal disulphide bridge linking strands β4 and β7; the unpaired cysteine (Cys287 in DDR1) is also buried and can be mutated without compromising DDR function [53]. The crystal structure is at variance with the suggestion that Cys303 and Cys348 mediate the covalent dimerisation of DDR1 [53]. The DS and DS-like domains of DDR1 are connected by a short linker and form a compact assembly, in which the two domains are related by a ~ 90° rotation. An extensive interface is formed between the flat bottom of the DS domain and the long insertion between strands β1 and β2 of the DS-like domain. Although the interface appears to be stable, it cannot be excluded that DDR activation is accompanied by substantial changes in the ectodomain arrangement, as seen in the epidermal growth factor receptor, for instance [54]. The DS-like domain of DDR1 is connected to the TM helix by a 50-residue JM region that is rich in proline and predicted glycosylation sites. The extracellular JM region of DDR2 is somewhat shorter (32 residues). These JM regions could plausibly form extended stalks that would project the globular heads of the DDR ectodomains a considerable distance (up to 150 Å for DDR1) from the cell surface.

The crystallisation of the DDR1 ectodomain was facilitated by the Fab fragment of mAb 3E3, which belongs to a series of anti-DDR1 mAbs that block the collagen-induced phosphorylation of DDR1 allosterically, i.e. without interfering with collagen binding [36]. The 3E3 Fab fragment is bound to the DS-like domain of DDR1, near the C-terminus of the crystallised construct. Two other blocking mAbs (1F7 and 1F10) also have their epitopes in this region, whilst three mAbs (3G10, 3H10 and 7A9) bind to a different epitope on the opposite face of the DS-like domain. The mAbs likely inhibit DDR1 activation by sterically hindering a conformational change or oligomeric association that is required for TM signalling.

## 5 Mechanism of DDR activation

It seems highly unlikely that the slow activation of DDRs in response to collagen [16,17] is solely due to conformational changes within the receptor or simple molecular association or dissociation events at the DDR cytosolic region. Rather, DDR activation might be limited by a cellular process, such as the redistribution of DDR (or another molecule essential for activation) in the cell membrane or their trafficking to another cellular compartment. DDR1 tagged with yellow fluorescent protein has been shown to be clustered within minutes of collagen binding [55]; it will be important to confirm this effect with untagged DDRs. Biochemical studies have shown that DDR2 activation requires the phosphorylation by Src of specific tyrosines in the activation loop of the DDR2 tyrosine kinase domain [56,57]; Src appears to be similarly involved in DDR1 activation [58]. Following phosphorylation by Src, the activated kinase domain of DDRs is believed to autophosphorylate several tyrosines in the JM region, which then recruit adaptor proteins such as Shc1 [17,56] and Nck2 [59], the protein tyrosine phosphatase SHP-2 [60], and members of the STAT family of transcription factors [60]. A recent proteomics study, in which DDR1b autophosphorylation was induced by pervanadate treatment (which inhibits protein tyrosine phosphatases), found three phosphorylated tyrosine residues within the JM region: Tyr484, Tyr513 and Tyr521 [61]. Three proteins were significantly enriched in immunoprecipitated activated DDR1b: the adaptors Shc1 and Grb2 and the RTK EphA2 [61]. Further experiments with phosphopeptides representing all possible tyrosine phosphorylation sites in DDR1 retrieved additional interactors, but it remains to be seen how many of them interact functionally with activated DDR1 in cells. The downstream signalling pathways activated by DDRs are not completely understood. Various studies have implicated the mitogen-activated protein kinase, phosphoinositide 3-kinase, Janus kinase and NF-kB pathways [4,5].

The central question for structural biologists is how ligand binding is converted into receptor activation. It was originally proposed that RTK activation is the result of ligand-induced receptor dimerisation, which brings the cytosolic tyrosine kinase domains into close proximity and thereby favours trans-autophosphorylation [2]. Many RTKs indeed seem to follow this scheme, but how ligands cause RTK dimerisation has turned out to be surprisingly variable [1]. Some RTK ligands are homodimers (e.g. nerve growth factor), providing a straightforward route to receptor dimerisation. Other RTK ligands are monomers (e.g. epidermal growth factor) and cause dimerisation by altering the structure of the receptor. However, the conceptually attractive mechanism of ligand-induced RTK dimerisation is complicated by the observation that many RTKs form non-covalent dimers even in the absence of a ligand [62–64].

Collagens form supramolecular assemblies with multiple binding sites for cellular receptors [18] and it is tempting to think that clustering by their multivalent ligands is what activates DDRs. However, there is little experimental evidence to support this mechanism. First of all, the oligomeric state of collagen in a typical activation experiment is poorly defined. Fibrillar collagens are soluble only at low pH, where they exist as isolated triple helices. Once added to the cell culture medium at neutral pH, the triple helices begin to aggregate into fibrils, and it is, therefore, difficult to know what oligomeric species activates the DDRs. An important experiment would be to test DDR activation by native collagen fibrils. The clearest evidence that the fibrillar state is not required for DDR activation comes from the observation that triple-helical collagen-like peptides containing the DDR-binding GVMGFO motif cause DDR phosphorylation with the same slow kinetics as a tissue-derived collagen [46,51]. This finding does not exclude that DDR clustering is important for activation, but it indicates that ligand multivalency is not essential.

Another important property of the DDRs is that they have a high propensity to form non-covalent dimers (or higher oligomers) within the cell membrane. Co-expression of differently tagged DDRs, followed by cell lysis and immunoprecipitation, demonstrated an association of the mature, cell surface-expressed, DDR glycoform, as well as of the intracellular biosynthetic precursors [65]. In these experiments, the level of co-immunoprecipitation was not affected by pre-treatment of the cells with collagen. A bacterial reporter assay (TOXCAT [66]) showed that the TM region of DDR1 has an intrinsic propensity to form dimers [65], and a subsequent comprehensive TOXCAT study found that the DDRs display the strongest TM interaction in the entire RTK family [64]. Collagen-independent DDR1 association has also been demonstrated by FRET microscopy [55]. Finally, the introduction of prolines into the TM region of DDR1 strongly impaired collagen-induced DDR1 activation, suggesting that specific TM interactions are important for DDR signalling [65].

**Fig. 4:**
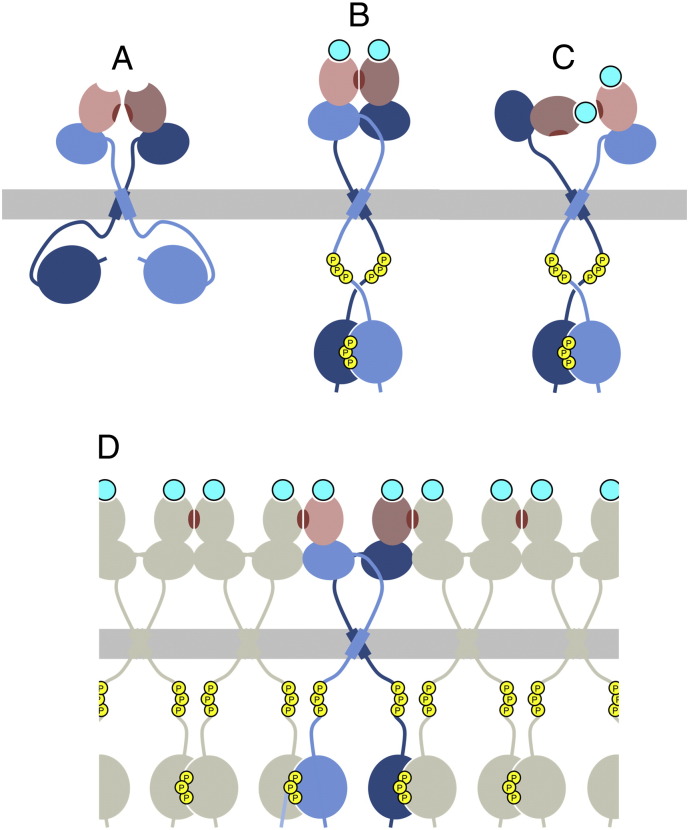
Possible mechanisms of DDR activation. (A) In the absence of collagen, inactive DDR is dimerised by interactions between the TM regions [65]. The long cytoplasmic JM region may be involved in DDR autoinhibition. (B) A collagen-bound active DDR dimer, in which the conserved patch in the DS domain (shown in brown) makes dimer contacts. Collagen triple-helices are shown as cyan filled circles. (C) An alternative active DDR dimer, in which the conserved patch contributes to collagen binding. (D) A cluster of collagen-bound DDR dimers, in which the conserved patch makes contacts between dimers.

How do the crystal structures of DDR ectodomain fragments fit into this picture? The DDR1 ectodomain lacking the JM region is monomeric in solution [36] and the complex of the DDR2 DS domain and a collagen-like peptide has 1:1 stoichiometry [37]. In co-crystals with the 3E3 Fab fragment, the DDR1 ectodomain formed a dimeric crystal lattice contact, in which a protruding loop from the DS-like domain of one molecule interacts with a highly conserved patch on the side of the DS domain of the neighbouring molecule [36]; this patch is distant from the collagen-binding site at the top of the DS domain (Fig. 3). Mutation of the protruding loop had no effect on DDR1 activation by collagen, suggesting that the crystal lattice dimer is unlikely to correspond to a signalling dimer on cells. In contrast, two mutations in the conserved patch (R32E, L152E) abolished DDR1 function, without affecting surface expression or collagen binding [36]. This result suggests that the conserved patch on the side of the DS domain is essential for DDR function either because it is involved in receptor-receptor contacts in the signalling complex or because it provides a weak secondary collagen-binding site (Fig. 4). Receptor–receptor contacts could be limited to a signalling dimer or lead to the formation of receptor clusters, similarly to the situation with Eph RTKs [67,68]. A putative secondary contact with collagen could be made by the other subunit in a DDR dimer or with another DDR dimer, thus forming collagen-linked DDR clusters (not shown). These hypotheses are testable in principle. The GVMGFO-containing collagen peptide uses only two of its three chains to bind to the DS domain [37] and the third chain is a likely candidate for a secondary contact. Thus, a collagen-like peptide with only two GVMGFO motifs may be unable to activate DDR on cells, even though it would still bind to the primary site at the top of the DS domain. The chemical synthesis of the required heterotrimeric collagen-like peptide is challenging, however. Furthermore, the interaction between DDR ectodomains and collagen-like peptides could be studied by analytical ultracentrifugation or electron microscopy and may reveal association states beyond a 1:1 complex. Conformational changes within the presumed DDR dimer could be studied by FRET microscopy [55]. However, to distinguish intramolecular conformational changes from receptor clustering, it will be essential to first determine the oligomeric state of DDRs at the cell surface, and how it is affected by collagen binding. This could be done using live cell single-molecule microscopy, which has already given invaluable insight into the activation mechanism of other RTKs [63].

Assuming that collagen somehow changes the conformation or oligomeric state of DDRs, how is this change translated into the activation of the cytosolic kinase domain? Given the unusually long JM regions at either side of the cell membrane, it is difficult to envisage a tight conformational coupling of the ligand-binding ectodomain and the cytosolic kinase domain [69]. The cytosolic JM regions of other RTKs contribute to autoinhibition of the kinase domains by a variety of mechanisms [70,71]. Frequently, autoinhibition is relieved by phosphorylation of tyrosine residues in the segment directly preceding the kinase domain [70]. There is no evidence that analogous proximal tyrosine residues are phosphorylated in activated DDRs; rather, the experimentally verified phosphotyrosines [61] are located ~ 100 residues N-terminal of the kinase domain (Fig. 1). Thus, if the cytosolic JM region is involved in DDR autoinhibition, as seems plausible, the mechanism is likely to differ from those described for other RTKs.

## 6 DDR mutations in human diseases

Mutations in the human *DDR2* gene cause a rare form of dwarfism, spondylo-meta-epiphyseal dysplasia short limb-hand type [28]. Three of the four identified missense mutations map to the kinase domain (T713I, I726R, R752C) and result in retention of the mutated proteins in the endoplasmic reticulum [27]. In contrast, the E113K mutant is normally expressed at the cell surface, but does not bind collagen [27]. This result is neatly explained by the crystal structure of a DDR2 DS-collagen complex, which shows that an Arg105-Glu113 salt bridge recognises the hydroxyproline in the GVMGFO motif (Fig. 2C).

The expression and activation status of DDRs is often increased in human cancers [5]. In addition, somatic mutations in the *DDR1* and *DDR2* genes have been found in cancer cells [72–75], but the functional consequences of the mutations have been studied only in a few cases. A recent study discovered several DDR2 mutations in primary lung squamous cell cancer (SCC) samples and cell lines [74]. A I638F mutation in the kinase domain of DDR2 was shown to promote the formation of cell colonies in soft agar, suggesting that the mutation may confer an oncogenic gain-of-function phenotype. The I638F mutant was also more sensitive to dasatinib inhibition than wild-type DDR2. Inspection of the homologous c-Abl kinase domain structure [76] shows that Ile638 in DDR2 is located at the back of the active site cleft, in the loop connecting helix αC to strand β4 (not shown). Thus, it is plausible that the I638F mutation could increase the basal activity of DDR2. Another DDR2 mutation, L63V, was also reported to promote the formation of cell colonies in soft agar [74]. Leu63 is conserved in all vertebrate DDRs and located close to the conserved surface patch on the side of the DS domain, ~ 10 Å from the functionally critical Leu152. How the L63V mutation could produce a hyperactive DDR2 is not clear; conceivably, the mutation could destabilise an autoinhibited form of the receptor. Finally, a R105S mutation in DDR2 was detected in a patient with large cell lung carcinoma [72]. Interestingly, Arg105 is a key residue in collagen recognition (Fig. 2C) and its mutation may, therefore, inactivate the receptor. Further functional studies will be important to determine whether the somatic DDR mutations found in cancer cells are causal with regards to oncogenesis.

## 7 Concluding remarks

Since the discovery of collagen as the DDR ligand 15 years ago, steady progress has been made in understanding the structural basis of collagen recognition and signalling by DDRs, but major questions remain. Single collagen triple helices can activate the DDRs in cell culture experiments, but how does this relate to DDR signalling in vivo? Are the DDRs activated by collagen fibres, which are abundant in many tissues? If so, how do cells interpret the sustained signal of tonically active DDRs? Alternatively, the DDRs may only be activated by isolated triple helices in tissues undergoing remodelling and repair. Another important question that needs to be addressed is the mechanism underlying the slow activation kinetics. Here, we expect new insights to come from the application of live cell imaging at single-molecule resolution. Finally, it is frustrating that the crystallographic characterisation of the DDRs has not been more informative about the mechanism of receptor activation. It would be gratifying if the reagents generated by these efforts (e.g. collagen peptides, function-blocking mAbs, signalling-deficient DDR mutants) could be used to unlock the remaining mysteries.
